# Whole-Genome Sequencing Analysis of *Chromobacterium piscinae* Strain ND17, a Quorum-Sensing Bacterium

**DOI:** 10.1128/genomeA.00081-16

**Published:** 2016-03-03

**Authors:** Kok-Gan Chan, Nina Yusrina Muhamad Yunos

**Affiliations:** Division of Genetics and Molecular Biology, Institute of Biological Sciences, Faculty of Science, University of Malaya, Kuala Lumpur, Malaysia

## Abstract

Here, we report the draft genome sequence of *Chromobacterium piscinae* strain ND17. This bacterium was isolated from a fresh water sample in Malaysia and exhibits quorum-sensing activity. This first draft genome of *C. piscinae* strain ND17 will pave the way to future studies of the quorum-sensing properties of this isolate.

## GENOME ANNOUNCEMENT

Bacteria communicate to control physiological activities such as virulence determinants, competence, symbiosis, antibiotic production, and biofilm formation (1). This bacterial cell-to-cell communication is known as “quorum sensing” (QS), a term first introduced by Fuqua and colleagues (2, 3). QS is achieved by generation and response of QS signaling molecules which refer to small, self-generated signal molecules (4). QS is a function that is population density dependent (5).

*Chromobacterium piscinae* is a Gram-negative, aerobic, rod-shaped, motile bacterium with violet pigmentation (6). In this study, we report the whole genome of *Chromobacterium piscinae* strain ND17. *C. piscinae* strain ND17 was isolated from a fresh water sample in Malaysia. Genomic DNA was extracted by using a MasterPure DNA puriﬁcation kit (Epicentre, Inc., Madison, WI, USA). Next, a NanoDrop spectrophotometer (Thermo Scientific, Waltham, MA, USA) and Qubit 2.0 fluorometer (Life Technologies, Carlsbad, CA, USA) were used to determine the quality of the DNA extracted. An Illumina MiSeq sequencer (Illumina, Inc., San Diego, CA, USA) was used to sequence the bacterium genome. An average coverage of 38.5-fold was obtained for the draft genome of 4,089,562 bp in 223 contigs with an *N*_50_ of 43,813. The paired-end reads were trimmed and *de novo* assembled with CLC Genomic Workbench version 5.1 (CLC Bio, Denmark). Prodigal (version 2.60) was used for gene prediction (7) and Rapid Annotation Subsystem Technology (RAST) was used for gene annotation (8). tRNA was predicted with tRNAscan SE version 1.21 (9) and rRNA with RNAmmer (10).

The G+C content of the *C. piscinae* strain ND17 genome is 62.6%. The total number of predicted genes is 3,916, of which 3,506 are protein coding genes with predicted function numbers, equivalent to approximately 90% of the total number of predicted genes. A total of 81 tRNAs, two copies of 5S rRNA, and a copy of each 16S rRNA and 23S rRNA were predicted for strain ND17. Based on the annotation result, the *C. piscinae* strain ND17 genome is comprised of 89 genes responsible for virulence, disease, and defense, where most of the genes are connected with antibiotics and toxic compounds resistance. This is the first finding reported on QS of *C. piscinae*. We hope this annotated genome of strain ND17 will be a valuable tool for better understanding the QS mechanism of *C. piscinae* strain ND17.

### Nucleotide sequence accession numbers.

This whole-genome-shotgun project has been deposited at DDBJ/EMBL/GenBank under accession no. JTGE00000000. The version described in this paper is the first version, JTGE01000000.

## References

[1] Miller MB, Bassler BL 2001 Quorum sensing in bacteria. Annu Rev Microbiol 55:165–199. doi:10.1146/annurev.micro.55.1.165. 11544353

[2] Fuqua WC, Winans SC, Greenberg EP 1994 Quorum sensing in bacteria: the LuxR-LuxI family of cell density-responsive transcriptional regulators. J Bacteriol 176:269–275. 8288518 10.1128/jb.176.2.269-275.1994 PMC205046

[3] Yunos NYM, Tan WS, Koh CL, Sam CK, Mohamad NI, Tan PW, Adrian T-G-S, Yin WF, Chan KG 2014 *Pseudomonas cremoricolorata* strain ND07 produces *N*-acyl homoserine lactones as quorum sensing molecules. Sensors 14:11595–11604.2 doi:10.3390/s140711595. 24984061 PMC4168423

[4] De Kievit TR, Iglewski BH 2000 Bacterial quorum sensing in pathogenic relationships. Infect Immun 68:4839–4849. doi:10.1128/IAI.68.9.4839-4849.2000. 10948095 PMC101676

[5] Liu YC, Chan KG, Chang CY 2015 Modulation of host biology by *Pseudomonas aeruginosa* quorum sensing signal molecules: messengers or traitors. Front Microbiol 6:1226. doi:10.3389/fmicb.2015.01226. 26617576 PMC4637427

[6] Kämpfer P, Busse HJ, Scholz HC 2009 *Chromobacterium piscinae* sp. nov. and *Chromobacterium pseudoviolaceum* sp. nov., from environmental samples. Int J Syst Evol Microbiol 59:2486–2490. doi:10.1099/ijs.0.008888-0. 19622653

[7] Hyatt D, Chen GL, LoCascio PF, Land ML, Larimer FW, Hauser LJ 2010 Prodigal: prokaryotic gene recognition and translation initiation site identification. BMC Bioinformatics 11:119. doi:10.1186/1471-2105-11-119. 20211023 PMC2848648

[8] Aziz RK, Bartels D, Best AA, DeJongh M, Disz T, Edwards RA, Formsma K, Gerdes S, Glass EM, Kubal M, Meyer F, Olsen GJ, Olson R, Osterman AL, Overbeek RA, McNeil LK, Paarmann D, Paczian T, Parrello B, Pusch GD, Reich C, Stevens R, Vassieva O, Vonstein V, Wilke A, Zagnitko O 2008 The RAST server: Rapid Annotation using Subsystems Technology. BMC Genomics 9:75. doi:10.1186/1471-2164-9-75. 18261238 PMC2265698

[9] Lowe TM, Eddy SR 1997 tRNAscan-SE: a program for improved detection of transfer RNA genes in genomic sequence. Nucleic Acids Res 25:955–964. doi:10.1093/nar/25.5.0955. 9023104 PMC146525

[10] Lagesen K, Hallin P, Rødland EA, Staerfeldt H-H, Rognes T, Ussery DW 2007 RNAmmer: consistent and rapid annotation of ribosomal RNA genes. Nucleic Acids Res 35:3100–3108. doi:10.1093/nar/gkm160. 17452365 PMC1888812

